# [Expression of Concern] *In vitro* and *in vivo* inhibition of tumor cell viability by combined dihydroartemisinin and doxorubicin treatment, and the underlying mechanism

**DOI:** 10.3892/ol.2026.15624

**Published:** 2026-04-28

**Authors:** Xiang Tai, Xiao-Bei Cai, Zhang Zhang, Rui Wei

Oncol Lett 12: 3701–3706, 2016; DOI: 10.3892/ol.2016.5187

Following the publication of the above paper, it has been drawn to the Editor's attention by a concerned reader that, regarding the images in Fig. 7 on p. 978 showing hematoxylin and eosin-stained images of the spleen, liver, kidney and heart sections of the mice employed in this study, the Liver/Control and Liver/DOX panels were strikingly similar in appearance.

The authors have been contacted by the Editorial Office to offer an explanation for this apparent anomaly in the presentation of the data in this paper, and we are awaiting their response. Owing to the fact that the Editorial Office has been made aware of potential issues surrounding the scientific integrity of this paper, we are issuing an Expression of Concern to notify readers of these potential problems while the Editorial Office continues to investigate this matter further.

